# Supplementary material for: The accuracy of nucleic acid amplification tests (NAATs) in detecting human intestinal nematode infections: A protocol for a systematic review and meta-analysis
Source: PLoS One. 2022 Dec 12;17(12):e0278920. doi: 10.1371/journal.pone.0278920 (PMC9744273; doi:10.1371/journal.pone.0278920)
Supplement: Attachment — Submitted filename: PLOS ONE. Reviewers comments.docx [file pone.0278920.s002.docx]

**Title:** The accuracy of PCR-based methods in predicting human intestinal nematode infections: a protocol for a systematic review and meta-analysis of the accuracy of available diagnostic tests.

**Comments**

This is a well thought out protocol, focusing on diagnostic techniques. It is well written with clear and concise methods which are adequate to realise the study goals. However, I wish to raise a few issues that the authors should consider before publication of the protocol.

1. From the study objective, and the narrative in the whole protocol, it is clear that the goal of the systematic review is to evaluate different diagnostic techniques/ approaches that are available for evaluation of human intestinal nematodes. However, the title of the study is more biased to PCR-based approaches which are not the primary focus of the study. I will suggest that the title is reviewed to capture the whole study and not part of the study.
2. In the objective (Abstract section), the authors states that the study will specifically focus on microscopic and PCR based approaches, however in the Eligibility criteria (index tests Page 7 and 8), they propose to include serology based assays. This is a key area in diagnosis of human nematodes and needs to come out well in the objectives and also in the title.

Thank you
