# Supplementary material for: The accuracy of nucleic acid amplification tests (NAATs) in detecting human intestinal nematode infections: A protocol for a systematic review and meta-analysis
Source: PLoS One. 2022 Dec 12;17(12):e0278920. doi: 10.1371/journal.pone.0278920 (PMC9744273; doi:10.1371/journal.pone.0278920)
Supplement: Attachment — Submitted filename: Response to reviewers.docx [file pone.0278920.s003.docx]

Response to reviewers : Title: **The accuracy of nucleic acid amplification tests (NAATs) in detecting human intestinal nematode infections: a protocol for a systematic review and meta-analysis**

To,

The Editor/Reviewers,

PLOS ONE

Date: September 22, 2022

Subject: Addressing Editor’s/Reviewers’ Comments for PONE-D-22-16144

We appreciate the editor's and the two reviewers' inputs on our protocol. To the best of our abilities, we have attempted to address each and every concern brought up by the academic editor and the two reviewers. We believe that all the issues are satisfactorily addressed.

Sincerely on behalf of all the authors

Prof Kosala Weerakoon

**Journal Requirements:**

1. Please ensure that your manuscript meets PLOS ONE's style requirements, including those for file naming. The PLOS ONE style templates can be found at
   <https://journals.plos.org/plosone/s/file?id=wjVg/PLOSOne_formatting_sample_main_body.pdf>

and  <https://journals.plos.org/plosone/s/file?id=ba62/PLOSOne_formatting_sample_title_authorsaffiliations.pdf>

***Our response – Revised as requested adhering to the style guidelines.***

2. Thank you for stating the following financial disclosure:

“This review is a part of an intestinal parasitoses community evaluation project funded by the National Research Council of Sri Lanka (NRC 20-118). However, this particular publication (review protocol) is not funded through this grant. The funder had and will not have a role in study design, data collection and analysis, decision to publish, or preparation of the manuscript."

At this time, please address the following queries:

a) Please clarify the sources of funding (financial or material support) for your study. List the grants or organizations that supported your study, including funding received from your institution. 
b) State what role the funders took in the study. If the funders had no role in your study, please state: “The funders had no role in study design, data collection and analysis, decision to publish, or preparation of the manuscript.”
c) If any authors received a salary from any of your funders, please state which authors and which funders.
d) If you did not receive any funding for this study, please state: “The authors received no specific funding for this work.”

Please include your amended statements within your cover letter; we will change the online submission form on your behalf.

***Our response - We have included the relevant details of the financial disclosure in the cover letter.***

**Reviewers' comments:**

***Our response to reviewer #* 1**

***We thank the reviewer for his kind comments and useful insights. We found them very helpful to improve our protocol for the systematic review.***

**Reviewer #1:** This manuscript describes a protocol to perform a systematic review, analysing the diagnostic accuracy of PCR-based methods for the detection of human intestinal nematodes. Taking into account the increasing use of nucleic acid amplification tests (NAAT’s) in the diagnosis of intestinal helminths, I think the authors have chosen a timely topic for their review. However, I see several flaws and inconsistencies in their approach.

1. First of all, what I understand from the methods section is that the Kato-Katz smear will be used as the reference test. Therefore, clarity should be given about the way the Kato-Katz has been performed in each study, i.e. how many smears have been examined, based on how many stool samples and were there appropriate quality control measures in place. Based on the described performance of the Kato-Katz smear examination the authors could rank each individual study included and give them a different weight in the overall analysis.

***Our response - As suggested, we have updated the data extraction phase of the Reference test in the methods section, to include information on the performance of the Kato-Katz, i.e.***

***number of stool samples taken from a subject for the performance of the test, the duration between each stool sample collection, number of smears examined from each stool sample, whether any modifications were done to the standard protocol, and the important details of the modifications done, quality control measures applied during the Kato-Katz procedure. Accordingly, when we are carrying out the data extraction of the selected studies, we will extract all of the above-mentioned details from the primary study. Based on the performance of the Kato-Katz smear examination of the primary study, we will rank each study and give them different weights in the overall analysis. (Line 241-247)***

2. Furthermore, for Strongyloides and Enterobius, it is not clear to me how the authors want to compare the PCR outcome with Kato-Katz data. For these two species other microscopy procedures (Baermann, tape-test) are more appropriate.

***Our response - We have added the Baermann test and the Graham Scotch tape tests as reference tests for strongyloidiasis and enterobiasis, respectively.* (*Line 159,160,247-252*)**

3. In addition the authors should be more specific about the NAAT’s included. Even if limiting to a real-time PCR format, each test is different, be it either in design and target selection, as well as in procedures of sample preparation and DNA extraction, the output variable producing, and most importantly, the level of appropriate quality control measures implemented. How to interpret these differences between publications is not clarified in this protocol. The description of Line 187-188 I find therefore far from complete. This lack of uniformity is not only a major struggle for the analysis of PCR performance, but also for serology.

***Our response - As proposed, we have revised the Index test section of the data extraction to include the information on the NAATs, i.e test design, target selection, the procedure of sample preparation and DNA extraction, output variables produced, whether appropriate quality control measures were applied during the procedure Depending on the performance of the NAATs analysis, we will rank them accordingly and give different weights during the overall analysis. ( Line 232-237)***

4. This brings me to another point. In the abstract I read: “We plan to analyse the efficacy of microscopic- and PCR-based approaches in detecting human intestinal nematode infections in different health care settings.” In the key-words I noticed “serology”, but nothing is mentioned in the abstract. Only from the search strategy (line 225) and description at line 181-186 I understand that several immune-diagnostic tests will be included as index test, including antibody tests and antigen tests. This sounds rather ambitious to me. The authors should be more clear what the relevance is of including immunodiagnostic test and how they want to summarise the different tests available. My advise is to leave the immunodiagnostic tests out of the analysis.

***Our response - As suggested, we have dropped the analysis of immunodiagnostic tests from the protocol. As a result, the exclusion of the serology from the abstract and objectives is now addressed. Accordingly, we have removed “serology” from the keyword list and the search string.***

5. Also for the different microscopy-based procedures included, the descriptions are not very clear to me. In my opinion the protocol would improve if the focus would be on a comparison between real-time PCR as the index test and the most optimal microscopy procedure as a reference test. Concerning microscopy, this is Kato-Katz for most STH species and species-dedicated procedures for Strongyloides and Enterobius

***Our response - As suggested, we have removed all the other microscopic diagnostic methods as index tests.***

6. Abstract: the proposed reference test is not described (as it is in line 138-140).

***Our response - The reference tests were described in the abstract as suggested. (line 34-37)***

7. Line 62: other *Ancylostoma* species should also be included.

***Our response – Revised as suggested (revised line 54)***

8. Line 226: ELIZA should be ELISA.

***Our response - Serology was removed from the protocol. Accordingly, ELISA was also removed from this line.***

9. Line 226: “immunofluorescent antibody” is not an appropriate search term.

***Our response - As serology was removed from the protocol, the immunofluorescent antibody was also removed from this line.***

10. Line 278: “The gold standard: As there is no universally accepted gold standard, we will use those in the published literature and the WHO recommended gold standard, which is Kato- Katz.” I suggest to rephrase this as follows: “Reference test: As there is no universally accepted “gold” standard, we will use the Kato -Katz as the reference test for the most common soil transmitted helminths, as this test is generally used in published literature and also recommended by the WHO as the most appropriate reference for these species.”

***Our response - This line was revised as suggested. ( revised line 238- 241)***

**Reviewer #2:** Title: The accuracy of PCR-based methods in predicting human intestinal nematode infections: a protocol for a systematic review and meta-analysis of the accuracy of available diagnostic tests.

**Comments**

This is a well thought out protocol, focusing on diagnostic techniques. It is well written with clear and concise methods which are adequate to realise the study goals. However, I wish to raise a few issues that the authors should consider before publication of the protocol.

***Our response to reviewer # 2***

***We thank the reviewer for appreciating our work and value the comments and suggestions given by the reviewer. Our responses to the comments are stated below.***

1. From the study objective, and the narrative in the whole protocol, it is clear that the goal of the systematic review is to evaluate different diagnostic techniques/ approaches that are available for evaluation of human intestinal nematodes. However, the title of the study is more biased to PCR-based approaches which are not the primary focus of the study. I will suggest that the title is reviewed to capture the whole study and not part of the study.

***Our response - As the first reviewer has suggested, we have taken serology out of our protocol as an index test. So we did not include any serology-related details in the abstract. Therefore, we feel that there is no necessity of amending the title.***

2. In the objective (Abstract section), the authors states that the study will specifically focus on microscopic and PCR based approaches, however in the Eligibility criteria (index tests Page 7 and 8), they propose to include serology based assays. This is a key area in diagnosis of human nematodes and needs to come out well in the objectives and also in the title.

***Our response - As stated above, we have taken serology out of our protocol as an index test. Thus, we did not include any serology-related information in the abstract and the objectives.***
