# Supplementary material for: The accuracy of nucleic acid amplification tests (NAATs) in detecting human intestinal nematode infections: A protocol for a systematic review and meta-analysis
Source: PLoS One. 2022 Dec 12;17(12):e0278920. doi: 10.1371/journal.pone.0278920 (PMC9744273; doi:10.1371/journal.pone.0278920)
Supplement: Attachment — Submitted filename: Response to Reviewers.docx [file pone.0278920.s004.docx]

Response to reviewers: Title: **The accuracy of nucleic acid amplification tests (NAATs) in detecting human intestinal nematode infections: a protocol for a systematic review and meta-analysis**

To,

The Editor/Reviewers,

PLOS ONE

Date: November 22, 2022

Subject: Addressing Editor’s/Reviewers’ Comments for PONE-D-22-16144

We appreciate the editor's and the reviewers' constructive suggestions on our revised protocol. We have attempted to address all the issues brought up by the academic editor and the reviewers. We believe that all the issues are satisfactorily addressed.

Sincerely on behalf of all the authors

Prof Kosala Weerakoon

**Journal Requirements:**

1. Please review your reference list to ensure that it is complete and correct. If you have cited papers that have been retracted, please include the rationale for doing so in the manuscript text, or remove these references and replace them with relevant current references. Any changes to the reference list should be mentioned in the rebuttal letter that accompanies your revised manuscript. If you need to cite a retracted article, indicate the article’s retracted status in the References list and also include a citation and full reference for the retraction notice.

***Response – The modifications we made to the reference section are listed below.***

***We have added new references numbering 21, 22, 23, 42 and 43 and accordingly references 39 and 41 of the previous list were removed to avoid repetition. We have not added any retraced papers.***

**Our response to reviewer # 1**

***We appreciate the reviewer's thoughtful remarks and insightful observations. They were incredibly useful to us in enhancing our systematic review protocol.***

***The line numbers we've provided correspond to those in the clean manuscript.***

Reviewer #1: I read the rebuttal and reviewed the changes made in the manuscript. In my opinion the authors responded well to all points mentioned by the reviewers. In particular I appreciate the more focused approach of the protocol.
Still, I have a number of technical comments. Most of these comments relate to the NAAT-procedures. The authors could be more precise in their wording and definitions at several points throughout the manuscript.

The line numbers mentioned refer to the manuscript including track changes.

1. Abstract (line 40): “… to more specialised high sequencing technologies such as the NAAT.” It is not clear to me what the authors try to say here. NAAT is a very broad definition of all tests related to nucleic acid amplification. Consequently “the” (?) NAAT is not an example of sequencing. More the other way around. Furthermore, the abbreviation NAAT is explained in the title. Still this should be repeated in the abstract (and in the introduction, see below).

***Response – We have explained the NAATs in the abstract and also in the introduction section (lines 32-36, 109-121)***

***Abstract (lines32-36)***

***“Molecular methods use NAA to amplify the deoxyribonucleic acid (DNA) or ribonucleic acid (RNA) fragments of the parasite to detect and determine its presence using different technologies (NAAT). They have increased the sensitivity of detection and quantitation of intestinal nematode infections, especially in low infection intensity settings.”***

***Introduction (lines 109 -121)***

***“NAATs are different molecular methods designed to amplify specific DNA or RNA fragments into a higher number of copies in order to determine and characterize their presence. Types of NAATs used for the diagnosis of intestinal nematode infections are polymerase chain reaction (PCR), including conventional single, nested, real-time, multiplex PCR and digital PCR, isothermal assays like loop-mediated isothermal amplification (LAMP) quantitative nucleic acid sequence-based amplification (QT-NASBA), strand displacement amplification (SDA), Recombinase polymerase amplification (RPA), Strand invasion based amplification (SIBA), Multiple displacement amplification (MDA), and more recently, cell-free DNA detection. They have substantially increased the sensitivity of detection and quantitation of intestinal nematode infections, especially in low infection intensity settings. They are also highly specific as they can help differentiate between closely related, morphologically identical, human and zoonotic species compared to microscopic techniques which could easily misidentify these parasites”***

2. Abstract (line 53): indeed, the details of the data bases are not so relevant for an abstract.

***Response – As suggested, we have removed the details of the databases from the abstract***

3. Objectives (lines 151-152): I agree that the objectives improved by using NAATs and not “PCR-based methods”. But at the introduction section the terminology of NAATs have not been well introduced. In line 121-122 PCR is introduced as a method, but nothing is mentioned about other NAAT-methods, including isothermal amplification procedures, such as LAMP.

***Response – We have introduced the word NAAT and provided details of the NAATs carried out in the diagnosis of human intestinal nematode infections. (lines 109-121)***

***Introduction (lines 109 -121)***

***“NAATs are different molecular methods designed to amplify specific DNA or RNA fragments into a higher number of copies in order to determine and characterize their presence. Types of NAATs used for the diagnosis of intestinal nematode infections are polymerase chain reaction (PCR), including conventional single, nested, real-time, multiplex PCR and digital PCR, isothermal assays like loop-mediated isothermal amplification (LAMP) quantitative nucleic acid sequence-based amplification (QT-NASBA), strand displacement amplification (SDA), Recombinase polymerase amplification (RPA), Strand invasion based amplification (SIBA), Multiple displacement amplification (MDA), and more recently, cell-free DNA detection. They have substantially increased the sensitivity of detection and quantitation of intestinal nematode infections, especially in low infection intensity settings. They are also highly specific as they can help differentiate between closely related, morphologically identical, human and zoonotic species compared to microscopic techniques which could easily misidentify these parasites”***

4. Inclusion criteria (lines 187-188): LAMP is one example of several isothermal amplification procedures. It is not clear to me why only LAMP is mentioned and no other methods.

***Response – As proposed, we have included other isothermal amplification methods to the inclusion criteria other than LAMP (lines172 and 173)***

***Inclusion criteria (lines 172 and 173)***

***“NAATs: Conventional single PCR, nested PCR, real-time PCR, multiplex PCR, and isothermal amplifications assays including LAMP, NASBA, SDA, RPA, SIBA and MDA”***

5. Search strategy (lines 237-238): again it is not clear to me why only “loop-mediated isothermal amplification” OR “LAMP” is included in the search terms for PubMed. I suggest to include the term “isothermal amplification” as well.

***Response – As suggested, we have included “isothermal amplification” to the search string (lines 206)***

6. Data extraction (lines 326-327): I appreciate the inclusion of a weight-factor, based on the quality of the used diagnostic procedures. But the description of how this is done is completely lacking.

***Response – We will use GRADE classification to give different weights to the studies based on the quality of the diagnostic procedure. In the methodological quality assessment section, we have introduced the term GRADE and given the details of the classification and how it will be utilized to give different weights to the studies based on the quality of the diagnostic procedure employed. (lines 233-239)***

***(Lines 233-239)***

***“We will employ the Grading of Recommendations Assessment, Development and Evaluation (GRADE) approach to evaluate the quality of the body of evidence. Accordingly, the body of evidence will be graded as high, moderate, low, or very low quality if data is available. Two reviewers will separately evaluate the body of evidence for each gradable outcome in accordance with the study limitations, consistency of results, directness of evidence, precision, and study design in order to arrive at a grading. Finally, we will put together a summary of the evidence table using the GRADE development tool.***

6. Data extraction (line 346-347): ”…the number of smears taken”. Please explain. I have never heard of “smears” in the context of a Baermann procedure. In addition ‘the standard technique” is not existing for the Baermann. Rather different approaches have been described.

***Response – We have removed “the number of smears taken” and “the standard technique” in the data extraction section of the Baermann test and included the “details of the different approaches of the Baermann test”.(lines 277 and 278)***
